# Digital Health Literacy in Bipolar Disorder: International Web-Based Survey

**DOI:** 10.2196/29764

**Published:** 2021-10-19

**Authors:** Emma Morton, Kendall Ho, Steven J Barnes, Erin E Michalak

**Affiliations:** 1 Department of Psychiatry University of British Columbia Vancouver, BC Canada; 2 Department of Emergency Medicine University of British Columbia Vancouver, BC Canada; 3 Department of Psychology University of British Columbia Vancouver, BC Canada

**Keywords:** eHealth, health literacy, bipolar disorder, self-management

## Abstract

**Background:**

Web-based resources can support people with bipolar disorder (BD) to improve their knowledge and self-management. However, publicly available resources are heterogeneous in terms of their quality and ease of use. Characterizing digital health literacy (the skillset that enable people to navigate and make use of health information in a web-based context) in BD will support the development of educational resources.

**Objective:**

The aim of this study was to develop understanding of digital health literacy and its predictors in people with BD.

**Methods:**

A web-based survey was used to explore self-reported digital health literacy (as measured by the e-Health Literacy Scale [eHEALS]) in people with BD. Multiple regression analysis was used to evaluate potential predictors, including demographic/clinical characteristics and technology use.

**Results:**

A total of 919 respondents (77.9% female; mean age 36.9 years) completed the survey. Older age (β=0.09; *P*=.01), postgraduate education (β=0.11; *P*=.01), and current use of self-management apps related to BD (β=0.13; *P*<.001) were associated with higher eHEALS ratings.

**Conclusions:**

Levels of self-reported digital health literacy were comparable or higher than other studies in the general population and specific physical/mental health conditions. However, individuals with BD who are younger, have completed less education, or are less familiar with mental health apps may require extra support to safely and productively navigate web-based health resources. Relevant educational initiatives are discussed. Future studies should evaluate skill development interventions for less digitally literate groups.

## Introduction

Self-management, the process of monitoring and responding to the signs, symptoms, and consequences of an illness [1,2], is central to living well with bipolar disorder (BD). To do this effectively, individuals require information about symptoms, quality of life impacts, treatments, and effective wellness strategies [3]. However, substantive barriers to accessing these resources exist. Individuals with BD experience delays of up to 8 years between symptom onset and diagnosis [4,5]. Availability of appropriate care is limited: 50%-65% of people with serious mental illnesses (SMI) such as BD report having received treatment in the previous year [6], and it is estimate that only 50% of patients in treatment for BD receive psychosocial services [7]. People with BD experience high rates of stigma [8], which can discourage help-seeking [9]. Finally, physical distancing measures implemented to mitigate the COVID-19 pandemic have introduced further obstacles to obtaining in-person care [10,11].

Web and mobile-based (ie, smartphone) educational materials and self-management supports (referred to collectively as eHealth) may be accessed by individuals independently of health care services, circumventing barriers to treatment [12,13]. Unsurprisingly, individuals with BD are increasingly turning to eHealth resources: up to 75% of people with BD use the internet as a source of information regarding their illness and treatment options [14-18], and the majority of individuals with SMI report a willingness to receive support for mental health needs delivered via a computer or smartphone [19,20]. However, using such sources is not without risk: they may be difficult to understand, contain inaccurate or irrelevant information, be developed to sell products and services, or compromise a user’s privacy [21].

The quality of existing, publicly available eHealth resources for BD is highly heterogeneous. While one review of top search-engine results for “bipolar disorder” and “manic depressive illness” found websites had reasonably accurate content [22], a different analysis found prominent websites were largely commercial in nature and of variable quality [23]. The latter study also noted that the ranking of websites in internet search results did not correlate with their quality appraisals. As internet search results are influenced by a number of factors in addition to credibility (including the presence of keywords, website popularity, and the user’s location/search history) the authors noted concerns that patients would be unlikely to identify high-quality offerings via casual browsing (ie, within the first 20 search results). Publicly available apps for BD share similar limitations: a review found the majority of these failed to provide evidence-based educational content, did not use validated screening measures, and did not address recommended core components of self-monitoring [24].

Digital health literacy, a construct related to (but incorporating aspects distinct from) health literacy, describes a set of competencies necessary to seek out, understand, appraise, and productively use eHealth resources [25]. This overarching construct is comprised of six core skills [26] including (1) traditional literacy (basic reading and writing skills), (2) health literacy (the ability to understand and act on health information, specifically), (3) information literacy (knowledge of how information is stored and how to search effectively), (4) scientific literacy (understanding of health research processes, limitations, and potential biases), (5) media literacy (the ability to think critically about media content, particularly source credibility and potential biases) and (6) computer literacy (the ability to access and use new technologies/software). A growing body of research has sought to describe the presence of this skillset (and the consequent need for educational interventions/alternative information delivery), particularly in underserved populations (eg, older adults, ethnic minorities, low-income groups, and rural communities) who may be unable to access face-to-face support with their health needs [27], as well as individuals with chronic health conditions who may turn to web-based resources for information and self-management support [28].

Digital health literacy skills are of clear importance for people living with BD, given the variable quality of web and app-based offerings for this condition. In addition, people with BD may experience specific challenges in identifying and using health information in digital contexts [21]: many experience cognitive difficulties (including problems with memory, attention, planning, problem-solving, and processing speed) that may impact their ability to search effectively or critically appraise the trustworthiness of web-based resources. Further, people with BD often have complex health questions that are not readily addressed by simple search strategies (eg, related to polypharmacy and comorbid conditions). Lack of eHealth literacy skills may have negative consequences for people with SMI, as they risk both using unhelpful/unsafe web-based resources, as well as failing to identify resources or tools with the potential to support their self-management. This potentially limits the reach of evidence-based eHealth interventions. Indeed, there is evidence to show that individuals with lower health literacy are less likely to adopt eHealth resources or perceive them as useful, while simultaneously overestimating the privacy protections offered by health apps [29]. While ideally, the onus for ensuring the quality of digital health resources would be on developers themselves or regulators, in practice the international web-based context and commercial interests of platforms that host information/tools present numerous barriers to institutional oversight. Similarly, while ideally clinicians would play a role in screening and recommending appropriate web-based resources for SMI, many find it difficult to keep abreast of the rapidly evolving web-based context. A recent survey of health care providers showed that the majority report lacking the confidence and knowledge to recommend apps to patients with BD [30]. It has been suggested that although people with SMI increasingly have access to smartphones, many lack the skills to use them effectively, such as navigating app stores and selecting safe and effective options [31]. While some efforts are underway to increase the ability of clinicians to identify relevant and safe digital mental health resources [32], the fact remains that at present, many people with BD are left to independently search for and screen health information on the internet.

Given the above, characterizing the presence of eHealth literacy skills and the factors that predict them is necessary to identify groups at risk of using poor-quality health information and support the development of targeted education materials. To our knowledge, levels of digital health literacy in people with BD have not been formally investigated. The present study aimed to (1) describe levels of digital health literacy and associated behaviors in people with BD and (2) explore predictors of digital health literacy.

## Methods

### Study Design

An overarching international, web-based, cross-sectional survey was conducted with the aim to investigate use of and attitudes towards apps amongst people with BD (survey items are presented in full in Multimedia Appendix 1). The present analysis focuses on responses to items concerning digital health literacy and associated behaviors. Use of apps is briefly summarized to contextualize the sample.

Questionnaires were administered via Qualtrics. Data collection occurred between February 19 and July 20, 2020. The study received ethics approval from the University of British Columbia Behavioral Research Ethics Board. Data in the study were treated confidentially and survey responses stored on a secure server in Canada. Participants received written information on the study and indicated their consent before proceeding.

### Participants and Recruitment

Participant recruitment was conducted with a combination of social media (Facebook, Instagram, and Twitter) advertising, Collaborative RESearch Team to study psychosocial issues in Bipolar Disorder (CREST.BD) email newsletters, and emails to health care providers or organizations associated with CREST.BD. Surveys were also advertised at a number of CREST.BD-hosted web-based (webinar) and in-person events for individuals with BD. Participants were offered the opportunity to be entered into a prize draw for 1 of 2 Can $50 (US $39.87) Visa gift cards. Inclusion criteria were (1) age ≥19 years and (2) a self-reported diagnosis of BD.

### Measures

#### Use of Apps

Individuals were asked to provide details about their frequency of use of apps in general, as well as use of apps specifically related to 2 core foci of self-management in BD (mood and sleep). Participants were asked to describe the sources of information they used to select apps; multiple options could be selected.

#### Digital Health Literacy

The e-Health Literacy Scale (eHEALS) was used to evaluate respondents’ perceived self-efficacy in identifying, applying, and evaluating the quality of digital health resources [33]. Eight self-report Likert-type items (1=“Strongly Disagree” to 5=“Strongly Agree”) are summed to create an overall score (range 8-40), with higher scores indicating greater knowledge and skills. Two additional Likert-type items (not included in the overall score calculation) are used to characterize respondents’ perception of the utility and importance of digital health resources. The unidimensional structure and reliability of the eHEALS has been demonstrated in the general population [33-35], as well as chronic physical and mental health conditions [36-38]. In the present sample, reliability of the scale was high (Cronbach α=.90).

To characterize self-reported confidence across specific competencies, Likert-scale ratings were simplified: the top 2 (“agree” and “strongly agree”) and bottom 2 (“disagree” and “strongly disagree”) options were collapsed to indicate “agree” and “disagree,” respectively.

### Data Analysis

Descriptive statistics were used to summarize survey responses. Multiple regression analysis was performed to evaluate the effects of demographic variables (age, gender, education level, and BD diagnosis) and app use behaviors (frequency of app use in general and use of BD-related health apps; ie, those designed to measure/support mood and sleep) on self-reported digital health literacy. Categorical variables were dummy-coded in reference to the following variables: gender (male), diagnosis (BD-I), education level (any high school), frequency of app use (less than daily), use of BD-related health apps (no). Prior to conducting regression analyses, appropriateness of eHEALS data for regression was confirmed via inspection of the Normal P-P plot, skew (–0.9), kurtosis (1.0), and Durbin-Watson statistics (2.0), variance inflation factors, and a plot of standardized residuals against predicted values. Statistical significance was set a *P*<.05. Data were analyzed using SPSS (version 26, SPSS Inc).

### Ethical Standards

The authors assert that all procedures contributing to this work comply with the ethical standards of the relevant national and institutional committees on human experimentation and with the 2008 revision of the Helsinki Declaration of 1975. All participants provided written informed consent.

### Availability of Data

Data are not publicly available in accordance with ethics approval given by the ethics board from the participating university. Interested investigators may submit inquiries to the corresponding author.

## Results

### Sample

A total of 919 people with BD responded to the web-based survey (see Table 1 for demographic/clinical characteristics and technology use behaviors). Overall, 81.3% of participants completed the survey between June 21 and July 20. The sample was primarily female (n=716, 77.9%), of White/European ethnicity (n=560, 61%), had a mean age of 36.9 (SD 12) years, and most commonly self-reported a diagnosis of BD II (n=477, 51.9%). The majority of the sample had completed some form of education beyond high school (n=551, 77.4%).

**Table 1:** Sample characteristics.

Demographic variable		Value
Females, n (%)		716 (77.9)
Age (years), mean (SD)		36.9 (12)
**Bipolar disorder diagnosis, n (%)**		
	Bipolar disorder I	321 (34.9)
	Bipolar disorder II	477 (51.9)
	Other bipolar disorders/No formal diagnosis	121 (13.2)
**Ethnicity, n (%)**		
	White	560 (61.0)
	Black/African	40 (4.4)
	Asian	152 (16.6)
	Middle Eastern	22 (2.4)
	Latin American	48 (5.2)
	Other or multiple ethnicities	96 (10.5)
**Education level, n (%)**		
	Any high school	177 (19.3)
	Postsecondary	214 (23.3)
	Undergraduate	324 (35.3)
	Postgraduate	173 (18.8)
	Other	31 (3.4)
**How often do you use apps?, n (%)**		
	Less than daily or not at all	76 (8.3)
	Up to 2 hours a day	268 (29.2)
	2-4 hours a day	297 (32.3)
	5 or more hours a day	278 (30.3)
**Use of bipolar disorder** **–** **related health apps, n (%)**		382 (41.6)
	Mood	228 (24.8)
	Sleep	242 (26.3)

### Use of Apps

**Figure 1:**
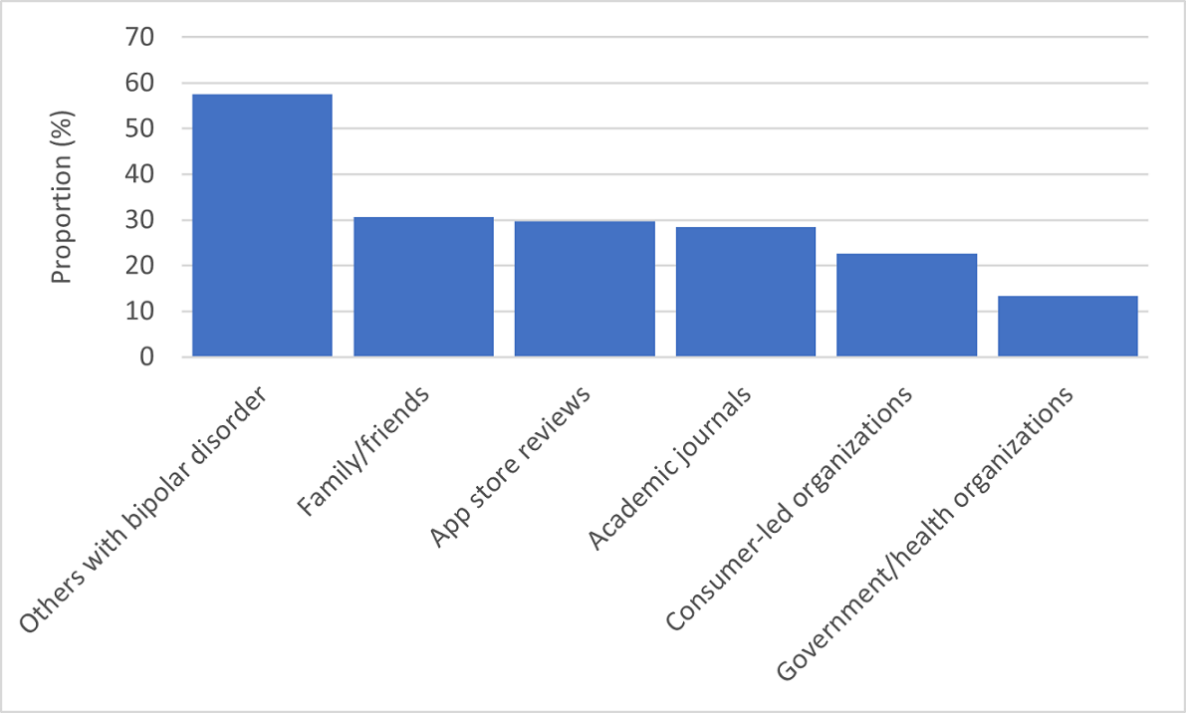
Preferred sources of information on health apps used by people with bipolar disorder.

Daily use of apps in general was reported by 91.7% (n=843) of the sample. A smaller proportion of respondents (n=382, 41.6%) endorsed using apps related to 2 core foci of self-management in BD: mood (n=228, 24.8%) or sleep (n=242, 26.3%). Respondents obtained information on health apps from a variety of sources (Figure 1); recommendations from other people with BD were commonly relied on (n=529; 57.6%), while government/health organizations were least commonly used (n=122; 13.3%).

### Digital Health Literacy

**Figure 2:**
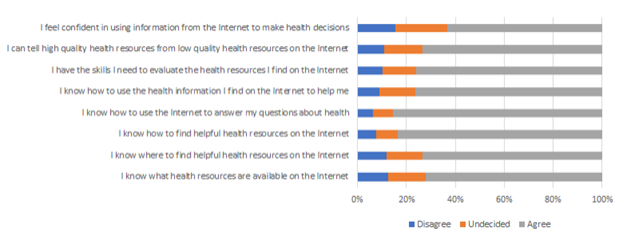
Endorsement of eHEALS (e-Health Literacy Scale) statements.

Participants regarded the internet as useful in making decisions about their health (mean 4.1, SD 0.8) and placed a high degree of importance on being able to access health resources on the internet (mean 4.4, SD 0.7). The mean level of self-reported digital health literacy as measured by the eHEALS was 31.7 (SD 6.3). Figure 2 illustrates the response frequencies for each eHEALS item; the majority of participants agreed with statements indicating they had the knowledge and skills to effectively search for, evaluate, and use web-based health information.

The regression model was statistically significant (*F*_11, 831_=4.2; *P*<.001), and explained 5.3% of the variance in eHEALS scores (Table 2). Of demographic variables, older age (β=0.09; *P=*.01) and postgraduate education (β=0.11; *P=*.01) were significant predictors of higher eHEALS scores. Other/unclear BD diagnosis was associated with significantly lower eHEALS scores (β=–0.11; *P=*.01). Finally, among variables describing current app use, only the use of apps related to BD was associated with significantly higher eHEALS scores (β=0.13; *P*<.001).

**Table 2:** Regression model predicting digital health literacy in bipolar disorder.

Variable		B (SE)	β	*t* test	*P* value
Age		0.05 (.02)	0.09	2.62	.01
Gender (female)		–0.05 (0.54)	–0.003	–0.09	.92
**Bipolar disorder diagnosis^a^**					
	Bipolar disorder-II	–0.56 (0.46)	–0.04	–1.21	.22
	Other bipolar disorder/no formal diagnosis	–2.45 (0.82)	–0.11	–3.0	.01
**Education level^b^**					
	Postsecondary	0.57 (0.66)	0.04	0.87	.39
	Undergraduate	0.90 (0.60)	0.07	1.50	.14
	Postgraduate	1.79 (0.70)	0.11	2.56	.01
**Frequency of app use^c^**					
	Up to 2 hours a day	0.66 (0.84)	0.05	0.78	.43
	2-4 hours a day	1.20 (0.84)	0.09	1.44	.15
	5 or more hours a day	0.83 (0.85)	0.06	0.98	.34
Use of bipolar disorder–related health apps		1.71 (0.44)	0.13	3.91	<.001

^a^Bipolar disorder diagnosis variables have the reference category: bipolar disorder-I.

^b^Education variables have the reference category: any level of high school.

^c^Frequency of app use variables have the reference category: less than daily/no use.

## Discussion

### Principal Findings

Individuals with BD are increasingly turning to web- and mobile-based resources to obtain information about the disorder and support self-management practices; however, concerns exist about their safety and credibility [21,24]. This survey of app use in BD offers encouraging findings regarding the ability of this group to identify, understand, appraise, and apply health information in a web-based context. Levels of digital health literacy in the sample were comparable to or higher than those in studies in the general population [38-41] and chronic physical health [36,38] and mental health conditions [37,38].

Of demographic variables, older age and postgraduate education (ie, master’s degree/PhD) were associated with self-reported digital health literacy in BD. The influence of higher education levels is replicated in a number of general population studies [27,39,41-43]. Although it may be expected that older age is associated with lower levels of familiarity and confidence with eHealth resources [27,39], there is evidence to suggest that the influence of younger age on digital health literacy is not observed in some physical illnesses [38,44-46]. Potentially, individuals with chronic health conditions by necessity have greater familiarity with digital health resources. Indeed, a longer duration of engagement with digital health interventions is associated with older age [47]. However, such findings must be interpreted cautiously in light of this sample’s relatively young mean age (mean 37 years, SD 12 years).

Across prior literature, the most consistent predictor of digital health literacy is the frequency of electronic device and internet use [27,34,41,44,45,48-50]. In this study, the frequency of app use was not associated with eHEALS scores, although the use of a BD-related self-management app (operationalized as apps used to support/monitor mood or sleep) predicted higher literacy. Similarly, one study found that the use of digital health resources specifically, not the time spent on the internet in general, predicts digital health literacy [27]. Future studies should test such potential mediators along with the directionality of these relationships, as it is unclear whether patients with lower digital health literacy draw on alternative information sources (eg, health care providers and peers) or whether increased use of digital platforms leads to higher knowledge and skills.

Finally, this study highlights the value of exploring condition-specific predictors of digital health literacy: not meeting diagnostic criteria for BD-I or -II (either owing to a diagnosis of BD not otherwise specified or lack of formal diagnosis) was associated with lower eHEALS scores. Potentially, this may be reflective of lower health literacy skills in general, as people need to navigate complex health care systems and medical insurance to receive appropriate care and diagnosis. Together, demographic, clinical, and behavioral variables explained only a small proportion of variance in self-reported digital health literacy in BD (5.3%); cognitive difficulties, complexity of health information needs, or lack of knowledge about BD are potential predictors that warrant investigation in future research [21].

Although our findings suggest that digital health literacy among people with BD is on par with that among the general population, we note that the web-based context is rapidly transforming in a way that further complicates the search for and evaluation of health information/resources. The dynamic and rapidly expanding mental health smartphone apps marketplace, for example, is particularly challenging to navigate: there are over 10,000 publicly available offerings [51], app store search algorithms lack transparency and may be influenced by paid advertising [52], turnover in apps is high [53], and app descriptions often make scientific claims regarding effectiveness despite lack of appropriate high-quality evidence [54,55]. Furthermore, apps collect large amounts of potentially identifying data that may be compromised by data breaches or sold to third parties; to make informed choices, users must be aware of and able to understand the implications of unclear or nonexistent privacy policies common to existing health and wellness apps [24,56,57].

A number of projects have been initiated to support people in selecting safe and credible apps for mental health concerns, including the development of a framework with which to appraise the quality, useability, data protections, and evidence base of an app [58]. This has been used as the foundation of a public, web-based database of app ratings [59] to support clinicians and patients in the selection of apps that best suit their mental health needs. Government and health organizations are similarly curating libraries of recommended apps; however, these resources lack public visibility and users often report unsatisfactory search experiences that prompt them to turn to commercial app stores [60]. Likewise, the present analysis found that among people with BD, government/research websites were least commonly endorsed as a source of information on health apps. These findings are in in line with those of studies suggesting that people primarily rely on word of mouth or app store ratings and reviews to identify and select mental health apps [61-63]. Further, our survey results suggest that first-hand experience of using digital tools to live well with BD lends credibility to app recommendations. This expertise may be formalized in the creation of “digital navigator” roles (a position often held by people with lived experience) in mental health clinics; such specialists could support patients to identify and use apps to support their recovery goals [64,65].

A second initiative to upskill patients in the technical and health literacy skills required to use mental health apps is the Digital Opportunities for Outcomes in Recovery Services (DOORS) group education program [31]. Skills taught range from basic smartphone functions (eg, accessing Wi-Fi, sending SMS text messages, and making calls), navigating the app store and downloading apps, to making informed decisions about health apps. The 4-week program was reported to numerically improve eHEALS scores in individuals with schizophrenia-spectrum diagnoses, although the significance of this change was not statistically tested owing to the small sample size. The DOORS curriculum is freely available to encourage health services, and peer support groups improve and expand on the program; web-based training modules will shortly be released [66]. Future studies should evaluate the efficacy of such programs in improving digital health literacy in BD populations. Furthermore, qualitative research is required to identify ways to tailor content to the specific needs, interests, and vulnerabilities among people with BD. For example, impulsivity and risk taking is characteristic of hypomanic/manic states in BD [67]; as such, modules may need to provide education and strategies addressing how evaluation of privacy/financial risks of health apps can be impacted by BD symptoms.

### Limitations

Limitations related to the sample were present. Participants self-reported a diagnosis of BD; diagnosis was not confirmed with a structured clinical interview, which may have allowed individuals who did not meet diagnostic criteria for BD to complete the survey. There is limited research to describe the clinical characteristics of people who self-identify as having BD, and as such, the generalizability of present findings should be interpreted with caution. However, we note that reassuringly, an analysis of a random sample (n=100) of people applying to join a BD case registry found that 93% had a lifetime Diagnostic and Statistical Manual of Mental Disorders (fourth edition) bipolar spectrum diagnosis as confirmed by a face-to-face structured clinical interview [68]. Additionally, the survey itself was web-based and primarily related to app use; the self-selected sample may have had higher levels of familiarity and interest in eHealth. Only a small proportion of respondents (n=10) reported using neither a smartphone nor tablet device; limiting our ability to draw inferences regarding the eHealth literacy of this subgroup. Research using paper-based surveys and nondigital methods of recruitment (eg, letters and face-to-face or telephone-based strategies) will provide valuable information regarding the eHealth literacy levels of people with BD impacted by the digital divide. Although a bias in favor of higher levels of eHealth literacy cannot be ruled out, we note that smartphone ownership is increasing amongst people with SMI [69], and rates of smartphone ownership in the present sample were comparable to those of another large-scale survey on BD [20].

Limitations to the measurement of digital health literacy should also be noted. First, eHEALS reflects perceived, rather than demonstrated knowledge and skills; in practice, these may have small to moderate correlations [45,70]. As such, there is a risk that digital health literacy levels reported by survey respondents may not translate to real-world behaviors. Clinicians should therefore remain curious and enquire about the kinds of digital health resources used by their patients and seek to promote credible offerings where available. Researchers should similarly consider dissemination plans to increase the visibility and uptake of evidence-supported digital health tools for BD. However, we note some complementary evidence from this study: a forthcoming analysis of the quality and safety of the most commonly used self-management apps (n=9) utilized by survey respondents in accordance with a standardized framework [71] found that these largely had appropriate data security measures, and half had evidence to support their efficacy at improving mental health outcomes in general population samples (E Morton, PhD, unpublished data, June 2021).

A second limitation related to measurement is that the eHEALS was developed in 2006, prior to widespread availability of smartphones and uptake of social networking. As such, it may not fully reflect how individuals access web-based health information in the present day. For example, the ability of this instrument to account for how social networking interacts with eHealth knowledge and behaviors has been questioned [72,73]; this is important to consider in the context of BD, where peer interactions are often characterized by seeking and sharing advice [74-76]. Despite limitations, eHEALS is the most widely used digital health literacy scale [28,73], and its use permits comparison with the wider literature. However, we note that conceptual and methodological advancements in the measurement of digital health literacy are ongoing [73], particularly in light of rapid changes to the web-based context, and as such, future research should reassess the presence of this construct as modernized measures are developed and validated in SMI populations.

Finally, it is important to consider potential impacts of the COVID-19 pandemic on familiarity and confidence with technology. The vast majority of respondents (81%) completed the survey between June and July 2020. By this stage, most countries worldwide had recommended or mandated some form of physical distancing; for people with BD, these measures may have increased their exposure to telepsychiatry or digital health resources [77]. As such, the eHEALS scores described in this sample may not be directly comparable to those in studies conducted prior to 2020. Research with contemporary samples is required to directly compare digital health literacy in BD to that in the general population.

### Conclusions

People with BD may need to seek out information or self-management supports on the internet to respond to new, changing, or ongoing symptoms, or in response to barriers to accessing treatment. However, the quality of existing web- and mobile-based resources is variable; digital health literacy is required to identify, understand, appraise, and use eHealth resources. The present large-scale, international survey offers reassuring findings, with self-reported digital health literacy levels in BD on par with or higher than that in community samples. Future studies should evaluate the concordance between self-reported digital health literacy and real-world applications of knowledge in people with BD, as well as the potential for educational interventions to support the skill development of less digitally literate groups, including those impacted by the digital divide.

## Appendix

Multimedia Appendix 1 Survey Items.

## Abbreviations

BD: bipolar disorder
CREST.BD: Collaborative RESearch Team to study psychosocial issues in Bipolar Disorder
DOORS: Digital Opportunities for Outcomes in Recovery Services
eHEALS: e-Health Literacy Scale
SMI: serious mental illnesses
