# Supplementary material for: Digital Health Literacy in Bipolar Disorder: International Web-Based Survey
Source: JMIR Ment Health. 2021 Oct 19;8(10):e29764. doi: 10.2196/29764 (PMC8564668; doi:10.2196/29764)
Supplement: Multimedia Appendix 1 [file mental_v8i10e29764_app1.docx]

Survey of App Use: Perspectives from people living with bipolar disorder

Q1 Which Gender do you identify with?

- Woman
- Man
- Another gender (please specify)
- Transgender/Trans
- Prefer not to say

Q2 Your age

Q3 Country of residence

- Canada
- United States
- Other country (please specify)

Q4 Marital Status

- Single
- In a committed relationship
- Common-law
- Other (please specify)
- Married
- Divorced
- Prefer not to say

Q5 How would you describe your ethnic background? (Check all that apply)

- Black, African, Caribbean
- East Asian
- Latin American
- Indigenous (First Nations, Métis, or Inuit)
- Other/Multiple (please specify)
- Native American
- Middle Eastern
- South Asian
- White/European

Q6 Highest level of education

- Did not finish high school
- High school
- Post-secondary diploma/certificate/associate's degree
- Undergraduate degree
- Master's degree
- Ph.D
- Other (please specify)

Q7 Which category best describes your employment status?

- Employed full-time
- Employed part-time
- Casual employment
- Unemployed
- Pension (please specify type)
- Student full-time
- Student part-time
- Volunteer
- Retired

Q8 Think of this ladder as representing where people stand in their communities/towns/cities. At the top of the ladder are the people who have the highest standing in their community/town/city. At the bottom are the people who have the lowest standing in their community/town/city. Please see the image below: 
  

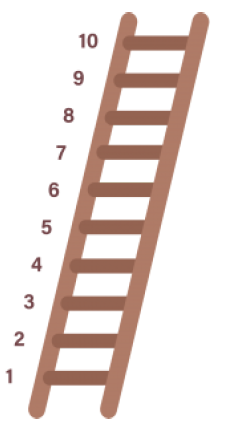

 
Where would you place yourself in this ladder?

- 1
- 2
- 3
- 4
- 5
- 6
- 7
- 8
- 9
- 10

Q9 Type of bipolar disorder

- Type I
- Type II
- No formal diagnosis received
- Other/don't know (please elaborate)

Q10 Are you currently in treatment for your bipolar disorder?

- Yes
- No

Q11 Which of the following treatment are you currently receiving?

- Medication(s)
- Counseling/psychotherapy
- Peer support
- Other (please specify)

**Current use of technology**
This section contains questions about your general use of technology and what features are important to you when using apps. The term "**app**" in this survey refers specifically to a mobile app - software application developed specifically for use on small, wireless computing devices, such as smartphones and tablets.

Q12 What device(s) do you use apps on?

- Smartphone
- Tablet
- Neither

Q13 Select the manufacturer of the primary device you use to access apps:

- Apple
- Samsung
- LG
- Google
- Huawei
- Xiaomi
- OnePlus
- Sony
- Unsure/prefer not to answer
- Other (please specify)

Q14 How often do you use apps on your smartphone/tablet?

- More than once a day
- Once a day
- Weekly
- Monthly
- A few times a year

Q15 On average, how many hours do you spend using apps on your smartphone/tablet a day?

- Up to 0.5
- 1
- 2
- 3
- 4
- 5 or more

**Current app use**In this section, we will be asking you about what kinds of smartphone apps you currently use to support your health and quality of life. We are particularly interested in the specific apps that are commonly used, so please try to provide the exact name of the apps you use. The term "apps" in this survey refers specifically to mobile apps - software applications developed specifically for use on small, wireless computing devices, such as smartphones and tablets.

Q16 Do you use any apps to help you incorporate exercise into your life?

- Yes
- No

Q16a Please list the exact name(s) of the apps you use to help incorporate exercise into your life. If you use more than one app, please separate the app names using a comma.

Q16b How often do you use apps to help incorporate exercise into your life?

- More than once a day
- Once a day
- Weekly
- Monthly
- A few times a year

Q17 Do you use any apps to help you manage your diet and nutrition?

- Yes
- No

Q17a Please list the exact name(s) of the apps you use to help you manage your diet and nutrition. If you use more than one app, please separate the app names using a comma.

Q17b How often do you use apps to help support diet and nutrition?

- More than once a day
- Once a day
- Weekly
- Monthly
- A few times a year

Q18 Do you use any apps to help manage your drug and alcohol use?

- Yes
- No

Q18a Please list the exact name(s) of the apps you use to help you manage your drug and alcohol use. If you use more than one app, please separate the app names using a comma.

Q18b How often do you use apps to help with managing drug and alcohol use?

- More than once a day
- Once a day
- Weekly
- Monthly
- A few times a year

Q19 Do you use any apps to help you optimize your sexual health and intimacy?

- Yes
- No

Q19a Please list the exact name(s) of the apps you use to help you optimize your sexual health and intimacy. If you use more than one app, please separate the app names using a comma.

Q19b How often do you use apps to help support sexual health or intimacy?

- More than once a day
- Once a day
- Weekly
- Monthly
- A few times a year

Q20 Do you use any apps to help you keep a routine in your sleep/wake cycle or improve the quality of your sleep?

- Yes
- No

Q20a Please list the exact name(s) of the apps you use to help you with sleep. If you use more than one app, please separate the app names using a comma.

Q20b How often do you use apps help support sleep?

- More than once a day
- Once a day
- Weekly
- Monthly
- A few times a year

Q21 Do you use any apps to track or manage your mood?

- Yes
- No

Q21a Please list the exact name(s) of the apps you use to track or manage your mood. If you use more than one app, please separate the app names using a comma.

Q21b How often do you use apps to track or manage your mood?

- More than once a day
- Once a day
- Weekly
- Monthly
- A few times a year

Q22 Do you use any apps to help improve your cognitive skills or manage problems with your thinking and memory?

- Yes
- No

Q22a Please list the exact name(s) of the apps you use to help support cognition or memory. If you use more than one app, please separate the app names using a comma.

Q22b How often do you use apps to help support cognition or memory?

- More than once a day
- Once a day
- Weekly
- Monthly
- A few times a year

Q23 Do you use any apps to help you keep up with your usual household chores, including keeping your home clean and organized?

- Yes
- No

Q23a Please list the exact name(s) of the apps you use to help with household management. If you use more than one app, please separate the app names using a comma.

Q23b How often do you use apps to help support household management?

- More than once a day
- Once a day
- Weekly
- Monthly
- A few times a year

Q24 Do you use any apps to help you maintain healthy self-esteem?

- Yes
- No

Q24a Please list the exact name(s) of the apps you use to help you maintain healthy self-esteem. If you use more than one app, please separate the app names using a comma.

Q24b How often do you use apps to help support self-esteem?

- More than once a day
- Once a day
- Weekly
- Monthly
- A few times a year

Q25 Do you use any apps to support your participation in leisure activities, hobbies, or creative pursuits?

- Yes
- No

Q25a Please list the exact name(s) of the apps you use to support your participation. If you use more than one app, please separate the app names using a comma.

Q25b How often do you use apps to support your participation leisure activities?

- More than once a day
- Once a day
- Weekly
- Monthly
- A few times a year

Q26 Do you use any apps to enhance your social life or relationships?

- Yes
- No

Q26a Please list the exact name(s) of the apps you use to support your social life and relationships. If you use more than one app, please separate the app names using a comma.

Q26b How often do you use apps to help support your social life or relationships?

- More than once a day
- Once a day
- Weekly
- Monthly
- A few times a year

Q27 Do you use any apps to support your spiritual practices or express your spirituality?

- Yes
- No

Q27a Please list the exact name(s) of the apps you use to support your spirituality. If you use more than one app, please separate the app names using a comma.

Q27b How often do you use apps to help support spirituality?

- More than once a day
- Once a day
- Weekly
- Monthly
- A few times a year

Q28 Do you use any apps to manage your finances?

- Yes
- No

Q28a Please list the exact name(s) of the apps you use to manage your finances. If you use more than one app, please separate the app names using a comma.

Q28b How often do you use apps to help support finances?

- More than once a day
- Once a day
- Weekly
- Monthly
- A few times a year

Q29 Do you use any apps to help you do your everyday activities independently (such as driving or using public transport)?

- Yes
- No

Q29a Please list the exact name(s) of the apps you use to help support you in living independently. If you use more than one app, please separate the app names using a comma.

Q29b How often do you use apps to help support your independence?

- More than once a day
- Once a day
- Weekly
- Monthly
- A few times a year

Q30 Do you use any apps to help you keep a stable, positive, sense of identity?

- Yes
- No

Q30a Please list the exact name(s) of the apps you use to help you keep a stable, positive, sense of identity. If you use more than one app, please separate the app names using a comma.

Q30b How often do you use apps to help support your identity?

- More than once a day
- Once a day
- Weekly
- Monthly
- A few times a year

Q31 Do you use any other apps to help manage your health and quality of life? Please list the exact app name(s). If you use more than one app, please separate the app names using a comma.

Q32 What are your favourite sources of information about health apps? Check all that apply:

- Other people with bipolar disorder
- Family or friends
- App store reviews
- Academic journals
- Organizations led by people with lived experience of bipolar disorder or other mental health conditions (please specify)
- Government or health organizations (please specify)
- Other (please specify)

Q33 Do you discuss your use of health apps with anyone? Check all that apply:

- Healthcare provider
- Family or friends
- Peer support settings (e.g., online forums or support groups)
- I don’t discuss my use of health apps with anyone
- Other (please specify)

Q34 Many factors can influence how we decide which app to use, and whether we continue to use it. Please indicate how important the following features are to you when considering health app for bipolar disorder:

|  | Not at all important | Slightly important | Moderately important | Very important | Extremely important |
| --- | --- | --- | --- | --- | --- |
| Quality/accuracy of its content |  |  |  |  |  |
| Interactivity (e.g., games or exercises) |  |  |  |  |  |
| Visually pleasing |  |  |  |  |  |
| Lets me personalize app features |  |  |  |  |  |
| App content is automatically tailored to my situation |  |  |  |  |  |
| Customizing the timing and frequency of notifications |  |  |  |  |  |
| Customizing what behaviours and feelings I keep track of |  |  |  |  |  |
| Ease of use |  |  |  |  |  |
| Medication or mood monitoring reminders |  |  |  |  |  |
| Flexibility to allow me to use the app in a way that works for me |  |  |  |  |  |
| Control over privacy and security of my information |  |  |  |  |  |
| Ability to talk to other people with bipolar disorder |  |  |  |  |  |
| Ability to share information with family/friends |  |  |  |  |  |
| Ability to share information with healthcare professionals |  |  |  |  |  |
| Feedback about how I am doing in an easy to understand format (e.g., graphs) |  |  |  |  |  |
| Flexible frequency of tracking (able to enter data multiple times a day) |  |  |  |  |  |
| Connectivity between different apps |  |  |  |  |  |
| Personalized coping and self-management strategies |  |  |  |  |  |
| Automatic collection of sleep or activity data based on phone use |  |  |  |  |  |
| Rewards for using the app |  |  |  |  |  |
| Free/low cost |  |  |  |  |  |
| Can be used without access to the internet |  |  |  |  |  |

Q35 Are there any other aspects of an app which would influence whether you would use it that are not addressed above?

Q36 Maintaining motivation to use an app over a long period of time can be a challenge. What features would an app need to have to make you want to continue using it?

**Interest and confidence in using eHealth resources**

We would like to ask you for your opinion and about your experience using the Internet for health information. For each statement, indicate which response best reflects your opinion and experience right now.

Q37 How useful do you feel the internet is in helping you make decisions about your health?

- Not useful at all
- Not useful
- Unsure
- Useful
- Very useful

Q38 How important is it for you to be able to access health resources on the internet?

- Not important at all
- Not important
- Unsure
- Important
- Very important

| Q39 | Strongly disagree | Somewhat disagree | Neither agree nor disagree | Somewhat agree | Strongly agree |
| --- | --- | --- | --- | --- | --- |
| I know how to find helpful health resources on the internet |  |  |  |  |  |
| I know how to use the internet to answer my health questions |  |  |  |  |  |
| I know what health resources are available on the internet |  |  |  |  |  |
| I know where to find helpful health resources on the internet |  |  |  |  |  |
| I know how to use the health information I find on the internet to help me |  |  |  |  |  |
| I have the skills I need to evaluate the health resources I find on the internet |  |  |  |  |  |
| I can tell high quality from low quality health resources on the internet |  |  |  |  |  |
| I feel confident in using information from the internet to make health decisions |  |  |  |  |  |

**Mobile Phone User Privacy Concerns**

| Q40 | Strongly disagree | Disagree | Somewhat disagree | Neither agree nor disagree | Somewhat agree | Agree | Strongly agree |
| --- | --- | --- | --- | --- | --- | --- | --- |
| I believe that the location of my mobile device is monitored at least part of the time |  |  |  |  |  |  |  |
| I am concerned that mobile apps are collecting too much information about me |  |  |  |  |  |  |  |
| I am concerned that mobile apps may monitor my activities on my mobile device |  |  |  |  |  |  |  |
| I feel that as a result of my using mobile apps, others know about me more than I am comfortable with |  |  |  |  |  |  |  |
| I believe that as a result of my using mobile apps, information about me that I consider private is now more readily available to others than I would want |  |  |  |  |  |  |  |
| I feel that as a result of my using mobile apps, information about me is out there that, if used, will invade my privacy |  |  |  |  |  |  |  |
| I am concerned that mobile apps may use my personal information for other purposes without notifying me or getting my authorization |  |  |  |  |  |  |  |
| When I give personal information to use mobile apps, I am concerned that apps may use my information for other purposes |  |  |  |  |  |  |  |
| I am concerned that mobile apps may share my personal information with other entities without getting my authorization |  |  |  |  |  |  |  |
